# Predicted 10-year risk of cardiovascular mortality in the 40 to 69 year old general population without cardiovascular diseases in Germany

**DOI:** 10.1371/journal.pone.0190441

**Published:** 2018-01-02

**Authors:** Claudia Diederichs, Hannelore Neuhauser, Viktoria Rücker, Markus A. Busch, Ulrich Keil, Anthony P. Fitzgerald, Peter U. Heuschmann

**Affiliations:** 1 Department of Epidemiology and Health Monitoring, Robert Koch Institute Berlin, Berlin, Germany; 2 German Center for Cardiovascular Research (DZHK), Partner Site Berlin, Berlin, Germany; 3 Institute of Clinical Epidemiology and Biometry, University of Würzburg, Würzburg, Germany; 4 Institute of Epidemiology and Social Medicine, University of Münster, Münster, Germany; 5 Department of Epidemiology and Public Health, Department of Statistics, University College Cork, Cork, Ireland; 6 Clinical Trial Center, University Hospital Würzburg, Würzburg, Germany; 7 Comprehensive Heart Failure Center Würzburg, University of Würzburg, Würzburg, Germany; Medizinische Universitat Innsbruck, AUSTRIA

## Abstract

**Aims:**

To estimate the 10-year risk of fatal cardiovascular disease (CVD) in the 40 to 69 year old general population in Germany stratified by sex and to analyze differences between socio-economic status (SES), region and community size in individuals without CVD. The analysis is based on the newly recalibrated SCORE Deutschland risk charts and considered other comorbidities for the classification of the high CVD risk group according to the guidelines of the European Society of Cardiology.

**Methods and results:**

In 3,498 participants (40–69 years) from the German Health Examination Survey for Adults 2008–2011 (DEGS1) without a history of CVD (myocardial infarction, coronary heart disease, heart failure, stroke) we estimated the proportion with a low (SCORE <1%), moderate (SCORE 1-<5%) and high 10-year CVD mortality risk (SCORE ≥5% or diabetes, renal insufficiency, SBP/DPB ≥180/110 mmHg or cholesterol >8 mmol/l). The prevalence of low, moderate and high risk was 42.8%, 38.5% and 18.8% in men and 73.7%, 18.1% and 8.2% in women. The prevalence of high risk was significantly lower in women with a high compared to a low SES (3.3% vs. 11.2%) and in communities with ≥100.000 inhabitants compared to <20.000 inhabitants (5.4% vs.10.9%). There were no significant associations between predicted CVD mortality risk and SES or community size in men and regions in men and women. Among the high risk group, 58.2% of men and 9.8% of women had SCORE ≥5%, leaving the majority of women (60.1%) classified as high risks due to diabetes and SCORE <5%.

**Conclusion:**

Our results suggest the persistence of socioeconomic disparities in predicted cardiovascular mortality in women and support the need of large-scale prevention efforts beyond individual lifestyle modification or treatment. Furthermore, the importance of additional comorbidities for the high risk group classification is highlighted.

## Introduction

Cardiovascular diseases (CVD) are the leading cause of death worldwide [1]. The risk of developing CVD is influenced by the single and combined effects of several factors, which can have high predictive values when they are combined in prediction models [2]. One of these models is the Systematic COronary Risk Evaluation (SCORE) project initiated in 2003 by the European Society of Cardiology (ESC) [3]. It was calculated as a function of age, sex, systolic blood pressure, smoking status and cholesterol level and estimates the absolute risk of fatal CVD within the next 10 years [4]. In order to guide preventive measure, the recent European Guidelines on Cardiovascular Disease Prevention in Clinical Practice defined different risk groups based on predicted risk with SCORE and on the prevalence of additional factors, for example comorbid diabetes [5].

Since 2003, the SCORE risk charts have been calibrated for several countries and regions to reflect national mortality and risk factor levels [4,6,7,8]. In Germany, the SCORE Deutschland risk charts were recently recalibrated on the basis of data from the population-based German Health Interview and Examination Survey for Adults 2008–2011 (DEGS1) and CVD mortality statistics from 2012 [8]. The need for an update arose from several trends including declining mortality rates for cardiovascular diseases, decreasing total cholesterol levels [9] and hypertension [10] and smoking prevalence [11] in the general population. Accordingly, the mean 10-year risk estimates for fatal cardiovascular diseases calculated with the recalibrated SCORE were almost one third lower compared to the older version and the percentage of high-risk individuals (with a SCORE ≥ 5%) declined from 5.2% to 2.6% when applied to the 40 to 65 year old DEGS1 population [8].

CVD morbidity or mortality risk prediction models are useful for risk assessment and communication at the individual level in order to guide lifestyle modification and treatment decisions [5]. In addition, they are a suitable tool for estimating future population trends in CVD morbidity and mortality [12]. Risk differences between population groups can be identified and used for targeted prevention measures. In Germany, specific CVD risk factors such as hypertension [13] or diabetes mellitus [14] show socio-economic and/or regional differences, but it is not clear if these differences also persist for overall CVD mortality risk based on the combination of multiple CVD risk factors.

According to the latest ESC guidelines [5], we applied the newly recalibrated SCORE risk charts to a sample of 40–69 year old participants of the latest national health examination survey in Germany DEGS1 without major CVD and classified as high risk those with an estimated 10-year risk of fatal cardiovascular disease (CVD) ≥ 5% or with diabetes, renal insufficiency, stage 3 hypertension or markedly elevated levels of cholesterol (> 8 mmol/l). The aim was to analyze overall CVD risk levels, differences between sexes as well as differences according to socio-economic status (SES), region and community size.

## Methods

### Study design and study population

**Fig 1:**
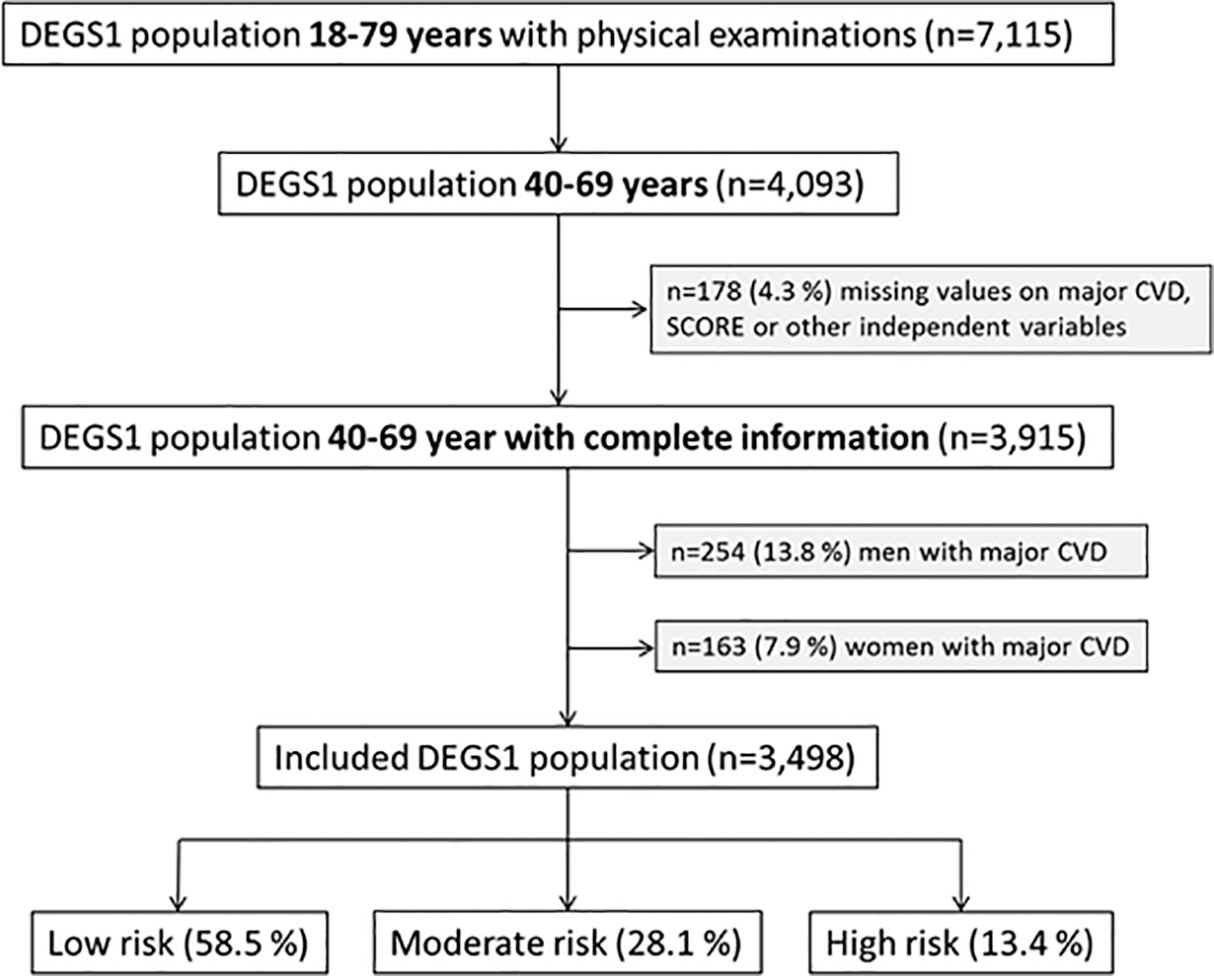
Selection of the study population.

All analyses are based on data from the German Health Interview and Examination Survey for Adults 2008–2011 (DEGS1). DEGS1 is a nationwide, population-based health survey of 7,987 adults between 18 and 79 years living in Germany. It includes former participants of the German National Health Interview and Examination Survey (GHNIES98) and newly sample individuals, thus allowing both cross-sectional and longitudinal analyses. More details are described elsewhere [15–17]. 7,115 out of 7,987 individuals had completed the physical examinations and 4,093 were aged between 40 and 69 years, i.e. in the age range in which risk prediction with the recalibrated SCORE is possible. After the exclusion of participants with missing data (n = 178) and with major CVD (n = 254 men and n = 163 women), the final study sample comprised 3498 individuals. More details are shown in Fig 1.

### Collection of information and definitions

Baseline data on age, sex, socioeconomic status (SES) and smoking status were collected in a self-administered questionnaire. The SES was measured with a multidimensional index based on education, occupational status and net equivalent income and was classified as low, medium and high [18]. Smoking was defined as current self-reported smoking [8]. Place of residence was used to derive information on region and community size of the participants.

Blood samples were taken at the beginning of the examination, processed within one hour and then restored at -40°C until analysis [17]. Serum concentrations of total cholesterol (TC) were determined using an enzymatic procedure (Architect ci8200, Abbott, Germany) [15]. TC levels >8 mmol/l (>310 mg/dl) were defined as markedly elevated [5]. Kidney function was determined from serum creatinine concentration (Architect, Abbott Diagnostics, Wiesbaden; IDMS traceable creatinine assay) and from cystatin C (Prospec, Siemens Healthcare, Eschborn) [19] and renal insufficiency was defined as a glomerular filtration rate (eGFR) <60 mL/min/1.73 m [5].

The second and third measurement of three consecutive automated blood pressure measurements with an oscillometric device (Datascope Accutorr Plus, Mahwah, NJ, US) were averaged [10]. Stage 3 hypertension was defined as a SBP/DPB ≥180/110 mmHg [5].

During a standardized, computer-assisted medical interview, participants were asked whether 1) myocardial infarction or chronic coronary heart disease, 2) heart failure, or 3) stroke had ever been diagnosed by a physician. Participants with at least one of the three diagnoses were classified as individuals with major cardiovascular diseases. Diabetes type 1 or type 2 was defined as either 1) self-reported, physician-diagnosed diabetes or taking oral anti-diabetic medication or insulin (ATC-A10) within the last seven days or 2) an HbA_1c_ ≥ 48 mmol/mol (≥ 6.5%) [14]. In the DEGS1 study sample, all individuals with antidiabetic medication had also diagnosed diabetes, which minimized the risk of misclassification. Only two individuals had diabetes type 1. Women reporting a diagnosis of diabetes during pregnancy, but no persistence of diabetes in the past 12 month and no current intake of anti-diabetic medication, were not counted as diabetic [20].

**Fig 2:**
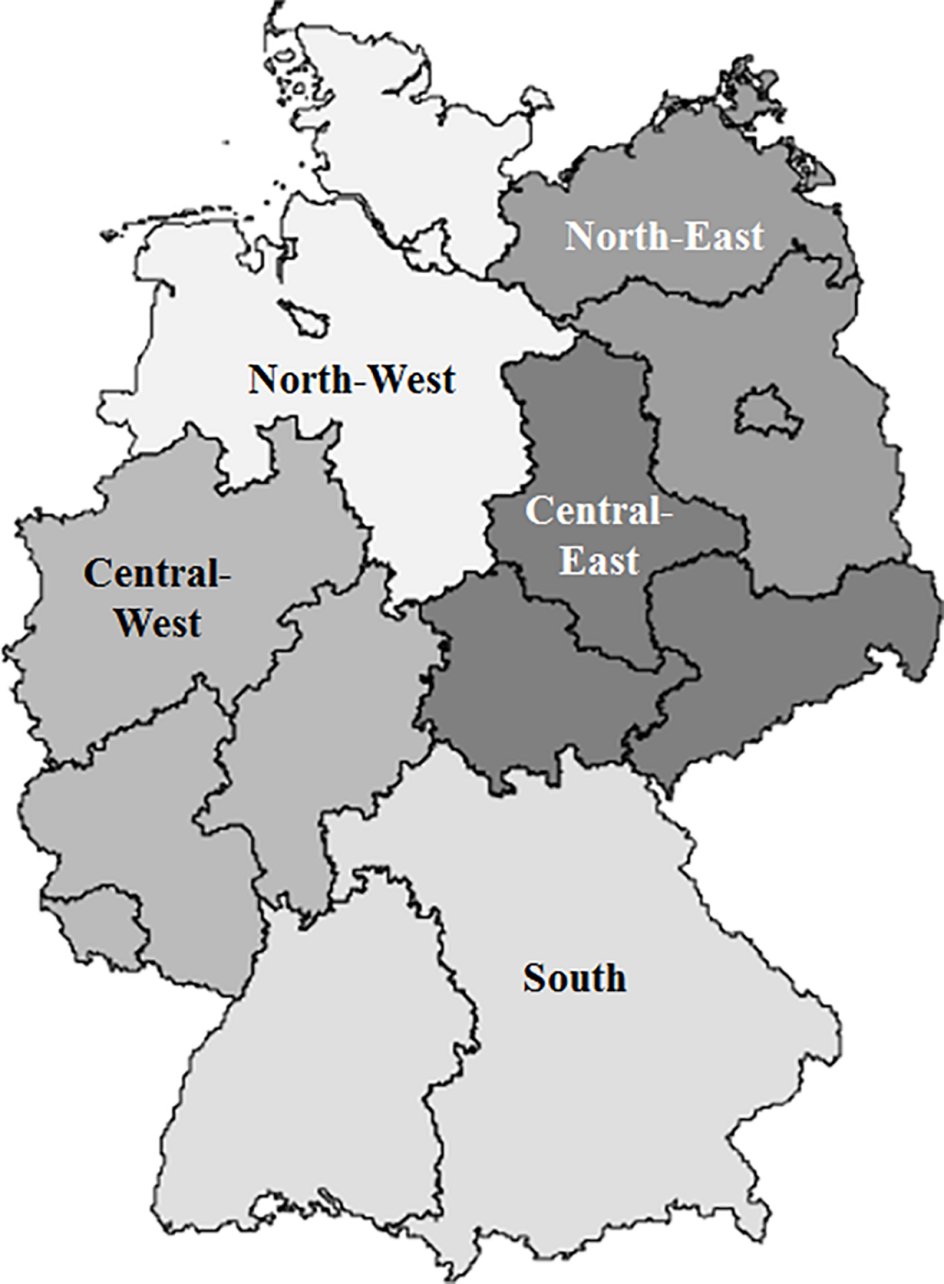
Overview on the five regions.

The 16 states in Germany were grouped into five regions considering the former inner-German border and by assigning three federal city states Berlin, Bremen and Hamburg consistently to their surrounding states (Fig 2).

### Calculation of the recalibrated SCORE and definition of risk categories

This study used the updated SCORE risk charts for Germany, based on total cholesterol (TC) levels (as opposed to the TC: HDL-C ratio), to estimate the absolute 10-year risk of fatal CVD in individuals without major CVD [3,8] in percent. A SCORE < 1% was defined as low risk, a SCORE from 1% to < 5% as moderate risk and a SCORE ≥ 5% as high risk. As recommended in the latest ESC Guidelines [5], we considered individuals with any of the additional comorbidities diabetes, renal insufficiency, stage 3 hypertension and markedly elevated levels of cholesterol (> 8 mmol/l) to be at high risk, regardless of their calculated SCORE risk. Therefore, the final risk categories were defined as follows: low risk (SCORE <1%), moderate risk (SCORE 1%—<5%) and high risk (SCORE ≥5% or any of the listed additional comorbidities).

### Data analysis

For all analyses, we used sample weights to adjust for the sampling design and correct for deviations between the study sample and the German population as of 31^st^ December 2010 with regard to age, sex, educational status, federal state, and type of municipality [16].

First, we quantified the percentage of participants with a low, moderate and high-risk for different age groups stratified by gender. Secondly, for each category of SES, region and community size, specified as independent variables, we determined the percentage of participants in the high risk group (SCORE ≥5% or any of the listed additional comorbidities), defined as the dependent variable. We used logistic regression models to analyze whether SES, region or community size had a significant influence on the odds to be classified as high risk. We both calculated unadjusted (Model 1) and adjusted odds ratios (Model 2), the latter was adjusted for the other two independent variables (SES, region or community size). We deliberately did not include any variables that were an integral component of SCORE, such as age, sex and smoking in the models to avoid over-adjustment. In sensitivity analyses, we calculated the mean and median SCORE (without additional risk factors) and adjusted Model 2 for age to see whether differences were only caused by other risk factors or variations in the age structure. Thirdly, we looked at the composition of the high risk groups and calculated the percentage of men and women who were classified as high risk persons in three steps: first due to SCORE ≥5%, then additional high risk cases due to diabetes and finally additional cases due to the other comorbidities (renal insufficiency, stage 3 hypertension, TC >8 mmol/l).

A p-value <0.05 was considered as level of statistical significance. All analyses were performed using Stata SE12 (StataCorp LP, Texas, US). Survey commands were used to account for the complex sampling design.

### Ethical considerations

Participants provided written informed consent prior to the interview and examination. The study was approved by the Federal and State Commissioners for Data Protection and the Charité-Universitätsmedizin Berlin ethics committee in September 2008 (No. EA2/047/08).

## Results

**Fig 3:**
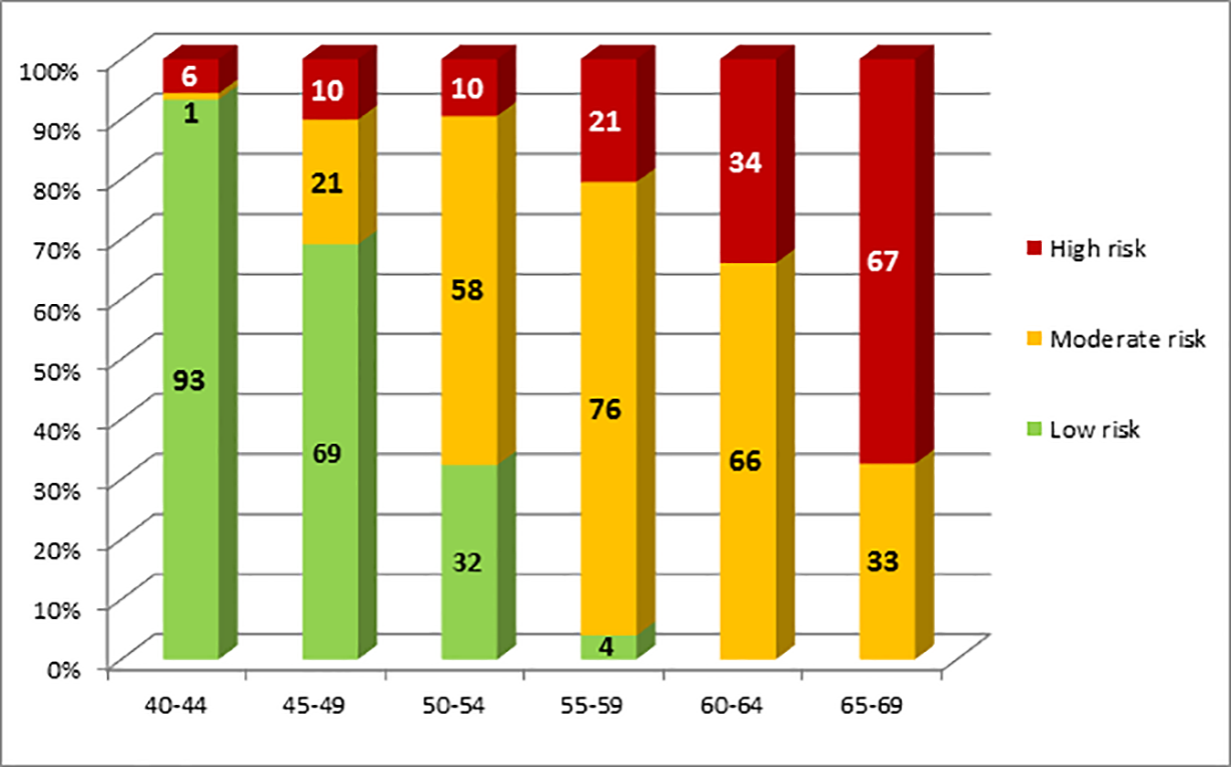
Percentage of 40–69 year old men without cardiovascular diseases with low, moderate and high 10-year CVD mortality risk* *low risk: SCORE<1%; moderate risk: SCORE 1-<5%; high risk: SCORE≥5% and/or any of the risk factors (diabetes, renal insufficiency, stage 3 hypertension or total cholesterol ≥ 8 mmol/l).

**Fig 4:**
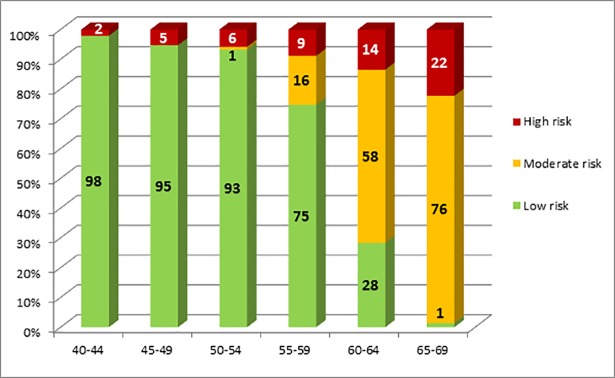
Percentage of 40–69 year old women without cardiovascular diseases with low, moderate and high risk 10-year CVD mortality risk* *low risk: SCORE<1%; moderate risk: SCORE 1-<5%; high risk: SCORE≥5% and/or any of the risk factors (diabetes, renal insufficiency, stage 3 hypertension or total cholesterol ≥ 8 mmol/l).

Characteristics of the study population are described in Table 1. Overall, 42.8% of men without major CVD had low risk, 38.5% moderate and 18.8% high risk of cardiovascular death within the next ten years. The respective numbers for women without major CVD were 73.7%, 18.1% and 8.2%. Age had a major influence on the risk for future CVD death. The percentage of individuals with a high risk increased from 5.7% in the youngest age group (40–44 years) to 67.4% in the highest age group (65–69 years) in men and from 2.1% to 22.3% in women (Fig 3 and Fig 4). In middle-aged (50–54 years) study participants, only one third (32.4%) of men had a low risk compared to 93.2% of women. In 55 to 59 old individuals, the percentage of men in the low risk group was 4.0% versus 74.8% in women.

**Table 1:** Characteristics of the 40–69 year old study population without cardiovascular diseases (n = 3,498, % are weighted population estimates).

		Men (n = 1,593)	Women (n = 1,905)
		%	%
**Sociodemographic variables**			
SES	Low	17.1	16.2
	Medium	57.1	62.8
	High	25.9	21.1
Region	South	28.7	28.3
	Central-West	34.0	35.1
	North-West	16.1	15.7
	North-East	9.7	9.3
	Central-East	11.5	11.6
Size of community	< 20,000	43.3	39.9
	20,000- < 100,000	27.1	31.3
	≥ 100,000	29.5	28.8
**Risk factors included in SCORE**			
Age	40–44	21.7	20.3
	45–49	22.3	21.3
	50–54	19.9	19.4
	55–59	15.1	15.9
	60–64	11.6	12.7
	65–69	9.4	10.4
Current smoking	Yes	24.9	21.5
Systolic blood pressure	Mean (mmHg)	129.1	122.4
Total cholesterol	Mean (mmol/l)	5.5	5.5
**Additional risk factors**			
Diabetes^1^	Yes	9.1	5.4
Renal insufficiency^2^	Yes	1.3	1.6
Stage 3 hypertension^3^	Yes	0.7	0.3
Total cholesterol ≥ 8 mmol/l	Yes	1.5	1.6
**Distribution of 10-year risk of cardiovascular death according to SCORE and additional risk factors**			
Risk^4^	Low	42.8	73.7
	Moderate	38.5	18.1
	High	18.8	8.2

1) HbA1c ≥ 6.5% and/or physician diagnosed diabetes and/or anti-diabetic medication (ATC A10), women with exclusively gestational diabetes were not counted as diabetic

2) GFR < 60 mL/min/1.73m^2^

3) SBP/DBP ≥ 180/110 mmHg

4) low risk: SCORE<1%; moderate risk: SCORE 1-<5%; high risk: SCORE≥5% and/or any of the risk factors (diabetes, renal insufficiency, stage 3 hypertension or total cholesterol ≥ 8 mmol/l)

Tables 2 and 3 show that in men, neither SES nor region or community size were significantly associated with the risk of future cardiovascular death. In women, the percentage of individuals with a high 10-year risk for cardiovascular death was significantly lower in individuals with a high socio-economic status (3.3%) compared to women with a low SES (11.2%) (p-value: 0.001). These differences remained significant after adjustment for region and community size (p-value: 0.004). Furthermore, women living in smaller communities with <20.000 inhabitants had a higher CVD risk, where 10.9% were in the high risk group compared to 7.4% of women living in cities with 20.000 -<100.000 inhabitants and 5.4% in larger cities with ≥100.000 inhabitants (p-value: 0.005). These differences remained significant after adjustment for SES and region (p-value: 0.008).

**Table 2:** Percentage of 40–69 year old men without cardiovascular diseases with a high 10-year CVD mortality risk* according to SES, region and community size.

		SCORE ≥5% and/or other risk factors	Model 1^1^		Model 2^2^	
		%	OR	p-value	OR	p-value
**Overall**		18.8	-	-	-	-
		(16.6–21.1)	-	-	-	-
SES	Low	19.8	Ref.	0.413	Ref.	0.496
		(14.9–25.8)	-		-	
	Medium	19.2	0.96		0.96	
		(16.3–22.5)	(0.66–1.41)		(0.65–1.40)	
	High	17.3	0.85		0.87	
		(13.7–21.5)	(0.55–1.30)		(0.56–1.33)	
Region	South	16.7	Ref.	0.497	Ref.	0.450
		(12.9–21.3)	-		-	
	Central-West	20.6	1.30		1.37	
		(16.6–25.3)	(0.87–1.94)		(0.91–2.05)	
	North-West	17.3	1.04		1.11	
		(12.6–23.2)	(0.65–1.68)		(0.70–1.77)	
	North-East	20.7	1.30		1.38	
		(14.6–28.5)	(0.78–2.19)		(0.83–2.29)	
	Central-East	19.1	1.18		1.18	
		(15.6–23.2)	(0.80–1.74)		(0.80–1.75)	
Size of community	< 20,000	20.1	Ref.	0.246	Ref.	0.247
		(16.5–24.2)	-		-	
	20,000- < 100,000	18.6	0.91		0.93	
		(14.8–23.2)	(0.63–1.31)		(0.64–1.34)	
	≥ 100,000	17.0	0.82		0.82	
		(14.0–20.6)	(0.59–1.14)		(0.59–1.14)	

*SCORE ≥ 5% and/or any other additional risk factor (diabetes, renal insufficiency, stage 3 hypertension or total cholesterol ≥ 8 mmol/l)

1) non-adjusted model

2) adjusted for all other risk factors (SES, region, size of community)

**Table 3:** Percentage of 40–69 year old women without cardiovascular diseases with a high 10-year CVD mortality risk* according to SES, region and community size.

		SCORE ≥5% and/or other risk factors	Model 1^1^		Model 2^2^	
		%	OR	p-value	OR	p-value
**Overall**		18.8	-	-	-	-
		(16.6–21.1)	-	-	-	-
SES	Low	11.2	Ref.	0.001	Ref.	0.004
		(6.9–17.7)	-		-	
	Medium	9.1	0.79		0.80	
		(7.4–11.1)	(0.44–1.41)		(0.45–1.43)	
	High	3.3	0.27		0.31	
		(2.0–5.6)	(0.12–0.58)		(0.14–0.66)	
Region	South	8.2	Ref.	0.157	Ref.	0.100
		(5.6–12.0)	-		-	
	Central-West	7.1	0.85		0.98	
		(5.2–9.6)	(0.50–1.44)		(0.57–1.66)	
	North-West	7.8	0.94		1.05	
		(4.7–12.5)	(0.48–1.84)		(0.55–2.01)	
	North-East	7.6	0.92		1.14	
		(3.9–14.4)	(0.41–2.10)		(0.51–2.58)	
	Central-East	12.8	1.63		1.61	
		(9.3–17.3)	(0.94–2.82)		(0.95–2.79)	
Size of community	< 20,000	10.9	Ref.	0.005	Ref.	0.008
		(8.5–14.0)	-		-	
	20,000- < 100,000	7.4	0.65		0.71	
		(5.5–9.7)	(0.43–0.98)		(0.46–1.07)	
	≥ 100,000	5.4	0.47		0.48	
		(3.4–8.4)	(0.27–0.81)		(0.27–0.85)	

*SCORE ≥ 5% and/or any other additional risk factor (diabetes, renal insufficiency, stage 3 hypertension or total cholesterol ≥ 8 mmol/l)

1) non-adjusted model

2) adjusted for all other risk factors (SES, region, size of community)

In sensitivity analyses, we found that the observed differences in women were also apparent in the distribution of the mean and median SCORE without including any additional risk factors. Therefore we concluded, that the differences between SES and community size in women were not caused by variations in the prevalence of additional risk factors. Furthermore, we showed that age-adjustment (Models 2) did not change the results in any significant way. We concluded that the observed differences in women were neither caused by variations in the age structure.

**Fig 5:**
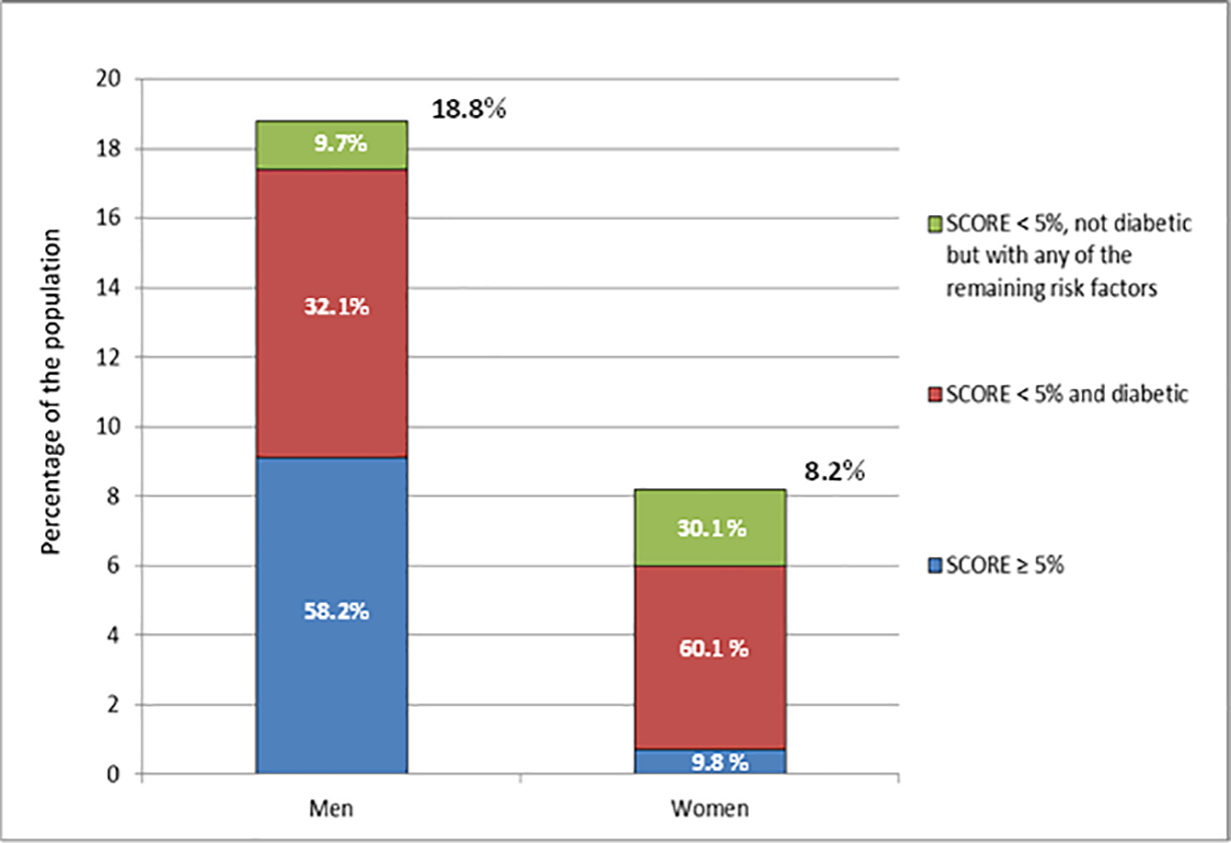
Contribution of single risk factors* to the high risk group in 40–69 year old men and women without cardiovascular diseases *Stepwise definition of the high risk group: First of all, the percentage of individuals with a SCORE ≥ 5% (including individuals with diabetes and other risk factors) were calculated, then the percentage of individuals with diabetes and finally, the percentage of individuals with any of the remaining risk factors (renal insufficiency, stage 3 hypertension or total cholesterol ≥ 8 mmol/l).

Fig 5 illustrates the contribution of a calculated SCORE ≥5% and of single additional risk factors (diabetes, renal insufficiency, stage 3 hypertension or TC >8 mmol/l) to the classification as a high risk individual stratified for men and women. In men, the majority (58.2%) of individuals belonged to the high risk category because of a SCORE ≥5%. Another 32.1% had diabetes and the remaining 9.7% had at least one of the other three comorbidities. However in women, this distribution was reversed. Only 9.8% of women were classified as high risk individuals because of a SCORE ≥5% and diabetes accounted for the majority of high risk cases (60.1%), followed by any other comorbidity (30.1%).

## Discussion

This study analyzed socio-economic and regional differences in estimated future cardiovascular mortality in adults without major symptomatic cardiovascular diseases in Germany. Over the entire age span from 40 to 69 years, the prevalence of individuals categorized as high risk was higher in men compared to women (18.8% versus 8.2%), while only 42.8% of men were classified as low risk versus 73.7% of women. For women only, a low socio-economic status and living in small communities with <20.000 inhabitants was associated with a higher risk for CVD death. Furthermore this study showed that among individuals with a high risk, 58.2% of men had a SCORE ≥5% in contrast to only 9.8% of women. Diabetes contributed nearly one third (32.1%) to the high risk group in men and 60.1% in women.

Cardiovascular risk prediction tools have been developed for risk stratification at the individual level and can have good predictive ability with high areas under the receiver operating curve in validation studies (e.g. SCORE validation studies have C-statistics of 0.62 to 0.91 [21]). However, they have rarely been used to provide valid estimates of future risk trends and disparities in the general population. The few existing population-based studies using SCORE are difficult to compare because of different national calibrations and exclusion criteria (e.g. in the Netherlands [7] individuals with a history of myocardial infarction were excluded, whereas in Switzerland [6] and in our study, individuals with a history of major CVD were not considered and other studies excluded individuals with diabetes [22], while we assigned them to the high risk group). Furthermore, studies using SCORE differ with regard to the definition of the high risk group. Most studies exclusively used a SCORE ≥ 5% as a selection criterion, whereas we extended the definition and also classified men and women with other comorbidities (diabetes, renal insufficiency, stage 3 hypertension and cholesterol levels >8 mmol/l) as high risk individuals, as proposed in the most recent ESC Guidelines [5]. This increased the size of the high risk group in our study substantially from 9.1% to 18.8% in men and even more pronounced, from 0.7% to 8.2% in women.

Overall, the percentage of men with a high 10-year risk for fatal CVD was about 2- to 3-times higher than in women in all age groups. Large predicted risk differences between sexes were also observed in the Netherlands where 8.5% of men and 0.8% of women had a SCORE ≥5% [7] and in Switzerland (15.8% versus 8.9%) [6]. The higher percentage of men in the high CVD risk category is in line with the consistently higher CVD mortality rates in the male population [23]. However, this may convey a misleading message since SCORE only applies to the 10-year CVD mortality risk in individuals aged 40 to 69, i.e. not to all ages and not to lifetime risk. For example, an analysis of the National Health and Nutrition Examination Survey 2003–2006 from the US data shows similar proportions of high lifetime risks among men and women 20 to 79 years old [24].

Another interesting result was that a low SES was significantly associated with a high 10-year risk of CVD death in women. These differences were also found in age-adjusted results and the mean and median SCORE, as part of the sensitivity analyses (data not shown). Of note, there is ample evidence that conventional CVD risk factors like the ones included in SCORE do not fully reflect social inequalities in CVD [25,26], and that CVD prediction scores have limited sensitivity to estimate CVD inequalities. Therefore the detected SES gradient in predicted CVD mortality in women is noteworthy and likely to be underestimated while in men SCORE may not be sensitive enough to detect a social gradient. In addition to this, a recent systematic review showed that different indicators of SES, such as education, occupation and area deprivation, were inversely associated with the risk of non-fatal and fatal cardiovascular diseases in both sexes, but the association was significantly stronger in women. Possible reasons include education-dependent sex differences in risk factors, in access and adherence to preventative treatment as well as treatment delays. However, the excess risk in women remained after adjustment for conventional CVD risk factors [27]. The authors concluded that sex-specific differences in the influence of SES on other CVD risk factors, such as diet, physical activity or BMI, which were not included in their models may partly explain the results. Other studies support this hypothesis [28,29].

We compared the percentage of the population with a high risk for CVD death between five different regions in Germany. This analysis complements a series of current studies with national survey data from the RKI, which showed that the highest age-adjusted prevalences of cardiovascular diseases [30] and the prevalences of several CVD risk factors such as physical inactivity, obesity, hypertension and diabetes [31] were found in the federal states in East Germany except for Berlin. In the present study, the percentage of women in the high risk group for future CVD mortality was slightly larger (12.8%) in Central-East compared to the other regions (all between 7.1% and 8.2%), but these differences were not statistically significant. The results for men were heterogeneous without any specific regional distribution. Adjustment for age, to consider age structure differences between regions, did not have a major influence on the results. Thus, the sensitivity of SCORE to detect regional differences on a population level may be limited and for example variations in regional health care provision are not considered. In our study, the percentage of women in the high risk group was significantly higher in small communities with <20.000 inhabitants. In Sweden, similar results were found for selected cardiovascular risk factors, where for example systolic blood pressure, BMI, total cholesterol levels and the prevalence of diabetes mellitus were significantly higher in the smallest communities [32]. These results were partly explained by the “healthy-migrant-effect”, where healthy and better educated individuals tend to move to more professional jobs in bigger cities. Along this line, the rural population in Sweden was older and less educated [33]. In contrast to this finding, no associations were found between the size of community and the incidence of hypertension in a prior analysis with GHNIES98 and DEGS1 data in Germany [13]. However, the variable community size has limitations since it is impossible to distinguish between small communities near a large city in contrast to sparsely populated areas which has a major influence on employment opportunities and infrastructure. Therefore, it is reasonable not to overestimate the role of community size in explaining cardiovascular risk differences.

Finally, we analyzed the composition of the high risk group in order to estimate the percentage of men and women who do not get classified as high risk by the calculated SCORE, but due to other comorbidities based on the recent ESC guidelines [5]. Data on this is scarce and it is remarkable that in the age group recommended for risk classification with the recalibrated SCORE (40–69 years), one in three men and nine in ten women do not get classified as high risk based on SCORE, but on other high risk conditions. This suggests that currently recommended CVD risk assessment and prevention tools might need further refinement by including additional comorbidities, particularly for women.

### Strengths and limitations

One major strength of this analysis was the use of national survey data with highly standardized measurements. Methodological differences between regions could thus be excluded. Continuous efforts were undertaken to maintain the response rate as high as possible throughout the study. Nevertheless, people with chronic diseases and reduced mobility are probably less likely to participate in the DEGS1 study and in its panel design is susceptible to intervention bias and regression to the mean effect. This may lead to an underestimation of the future cardiovascular risk in our study population. On the other hand, SCORE was recalibrated based on German mortality statistics [4,8], which include people with CVD who have a higher 10-year mortality risk. Thus, SCORE probably overestimates the CVD mortality risk among individuals with no history of CVD, especially in older subjects, where the prevalence of CVD is high.

Another strength of this study is the inclusion of additional risk factors in the classification of the high risk population in our analyses. However, this can only be used for classifying the high risk group and not for estimating the absolute risk. For the future, it would be desirable to develop risk charts that do not require such additional rules for classification of high risk, which are less evidence-based than the risk charts. Then, sex- and age-specific variations in the effect of comorbidities on cardiovascular risk must be considered, for example women with diabetes have a higher CVD mortality rate than men [34].

It is important to mention that in our analysis, we focused on at very specific study population between 40 and 69 years old without any physician diagnosed cardiovascular diseases, which might explain the sex-specific differences in the prevalence of diabetes. As a more general critique, although SCORE is widely used, there are several major shortcomings: risk prediction is limited to fatal CVD cases, which ignores all other common consequences of CVD such as long-term impairments, disability and a reduced quality of life; risk cannot be estimated for those older than 69 years although the burden of CVD is very large at older ages; and hypertension and hyperlipidemia treatment as well as diabetes status are not considered.

## Conclusions

Today the SCORE risk prediction model is a frequently used instrument to detect individuals with a high cardiovascular risk and to guide intensive lifestyle advice and drug treatment if necessary. We could show in this study that prediction models can also be a valuable tool for population health monitoring, since they combine weighted prognostic information from several risk factors into an estimation of future outcomes. Thus, they can detect potential future disparities between population groups. For example, our results suggest that special attention should be paid to SES differences in CVD mortality risk in women in Germany. Furthermore, it was a surprising finding that when applying the most recent ESC Guidelines [5] to a general population sample, high risk status was very frequently assigned due to single comorbidities (despite a SCORE<5%). Therefore, we believe that these criteria for high risk status should be highlighted more and their evidence-base should be documented in the guideline.
